# Acute airway eosinophilic inflammation model in mice induced by ovalbumin, house dust mite, or shrimp tropomyosin: a comparative study

**DOI:** 10.3389/falgy.2025.1594028

**Published:** 2025-06-03

**Authors:** Liangyu Xu, Zichen Wei, Rongfang Wu, Siqi Kong, Jinlian Bin, Yuxin Gao, Lei Fang

**Affiliations:** ^1^ Institute of Translational Medicine, School of Medicine, Yangzhou University, Yangzhou, China; ^2^ Pathology Department, Dongtai Hospital of Traditional Chinese Medicine, Yancheng, China; ^3^ Pharmacy Department, Yixing Hospital of Traditional Chinese Medicine, Wuxi, China; ^4^ Guangxi Key Laboratory of Reproductive Health and Birth Defects Prevention, Maternal and Child Health Hospital of Guangxi Zhuang Autonomous Region, Nanning, China; ^5^ The Key Laboratory of the Jiangsu Higher Education Institutions for Nucleic Acid & Cell Fate Regulation, Yangzhou University, Yangzhou, China

**Keywords:** ovalbumin, house dust mite, shrimp tropomyosin, airway inflammation, chemokine

## Abstract

**Background:**

Ovalbumin (OVA) and house dust mite (HDM) are widely used allergenic proteins in murine models of allergic asthma. In our previous studies, shrimp tropomyosin (ST) was shown to induce type I hypersensitivity, including asthma-like responses. Here, we compared airway eosinophilic inflammation models induced by OVA, HDM, or ST using a protocol of three intraperitoneal (i.p.) sensitizations followed by a single intratracheal (i.t.) allergen challenge.

**Methods:**

C57BL/6J mice were sensitized via three i.p. injections of OVA, HDM, or ST mixed with Al(OH)_3_, followed by a single i.t. challenge with the respective allergen. Lung transcriptomic analysis, plasma IgE levels, bronchoalveolar lavage (BAL) fluid cell counts, cytokine and chemokine mRNA levels, and histopathological assessments were performed to evaluate airway inflammation.

**Results:**

A single i.t. challenge with ST or HDM significantly increased the lung-to-body weight ratio, eosinophil infiltration, and mucus hypersecretion, accompanied by elevated mRNA levels of Th2 cytokines (*Il-4*, *Il-5*, *Il-13*) and increased the total cell count and eosinophil count in the BAL fluid. In contrast, OVA induced only mild eosinophilic inflammation, suggesting that repeated exposures may be required to elicit a robust allergic response. RNA sequencing and qRT-PCR further identified key chemokines associated with eosinophil recruitment (*Ccl-11*, *Ccl-24*), Th2 polarization (*Ccl-17*), and neutrophil activation (*Cxcl-1*).

**Conclusion:**

A single i.t. challenge of ST, similar to HDM, exhibits a potent ability to induce eosinophilic inflammation and Th2-type immune responses in a murine model of allergic asthma, surpassing the effects of OVA.

## 1 Introduction

Asthma, an allergic airway disease, is triggered by the inhalation of allergens. These allergenic proteins include food allergens (1) and environmental aeroallergens (2). Animal models should replicate key features of human asthma, such as airway hyperresponsiveness, inflammation, and airway remodeling, thereby providing valuable insights into the pathophysiology of the disease. Allergic asthma animal models are typically established through systemic sensitization and airway challenge with ovalbumin (OVA) or house dust mite (HDM). OVA is widely used in numerous experiments due to its availability in large quantities at a relatively low cost. Additionally, standard commercial OVA-specific antibody testing kits and OVA-specific transgenic mice offer reliable and convenient tools for research applications. However, OVA has certain limitations, including its lack of relevance as an aeroallergen in human asthma research and the requirement for adjuvants to sensitize animals (3). Despite requiring complex extraction processes, HDM models are widely regarded as the most clinically representative experimental system for allergic asthma research. Unlike OVA, HDM extracts contain a complex mixture of allergenic proteins (e.g., Der p 1 and Der p 2) that can sensitize animals through airway exposure without adjuvants (4). By mimicking key pathological features of human allergic asthma - including protease-mediated epithelial barrier disruption (5) and TLR4-dependent innate immune activation (6), HDM models have become indispensable for investigating asthma pathogenesis and evaluating therapeutic interventions.

Shrimp, belonging to the eight big food allergens (cow's milk, egg, wheat, soy, peanut, tree nuts, fish and shellfish) (7), contains tropomyosin as its predominant allergen. Tropomyosin is involved in cross-reactivity among mites and crustacean (8, 9). Critically, HDM allergens may serve as the initial sensitizing agent for shrimp allergy through IgE cross-reactivity (10). This bidirectional relationship is clinically significant: patients co-sensitized to both tropomyosins (Pen a 1 and Der p 10) exhibit exacerbated HDM-induced asthma (11). Thus, shrimp allergy is not merely a food allergy but a potential comorbidity and aggravating factor in aeroallergen-driven asthma, justifying its use in modeling airway inflammation. In our previous study, shrimp tropomyosin (ST) was used to induce asthma in mice through a protocol involving three sensitization sessions followed by 4–7 challenge exposures (12–14). We demonstrated that shrimp tropomyosin (ST) induces more robust allergic responses than ovalbumin (OVA) in murine models, even without adjuvant use during sensitization. This study revealed ST's unique capacity to bypass adjuvant requirements for Th2 sensitization—a critical advantage over OVA, which strictly depends on adjuvants to prime allergic responses (13). The current study shifts focus to compare the acute inflammatory potency of three allergens—OVA (a conventional food-derived model antigen), HDM (a natural aeroallergen), and ST (a clinically relevant food allergen with cross-reactive potential) under adjuvant-assisted sensitization followed by a single intratracheal (i.t.) challenge. This study aims to addresses whether ST retains its superior immunogenicity over OVA under acute challenge conditions, and whether a single i.t. allergen exposure suffices to elicit HDM-like eosinophilic inflammation, bypassing the need for prolonged challenge protocols.

## 2 Methods

### 2.1 Airway inflammation model induced by different allergens

**Figure 1:**
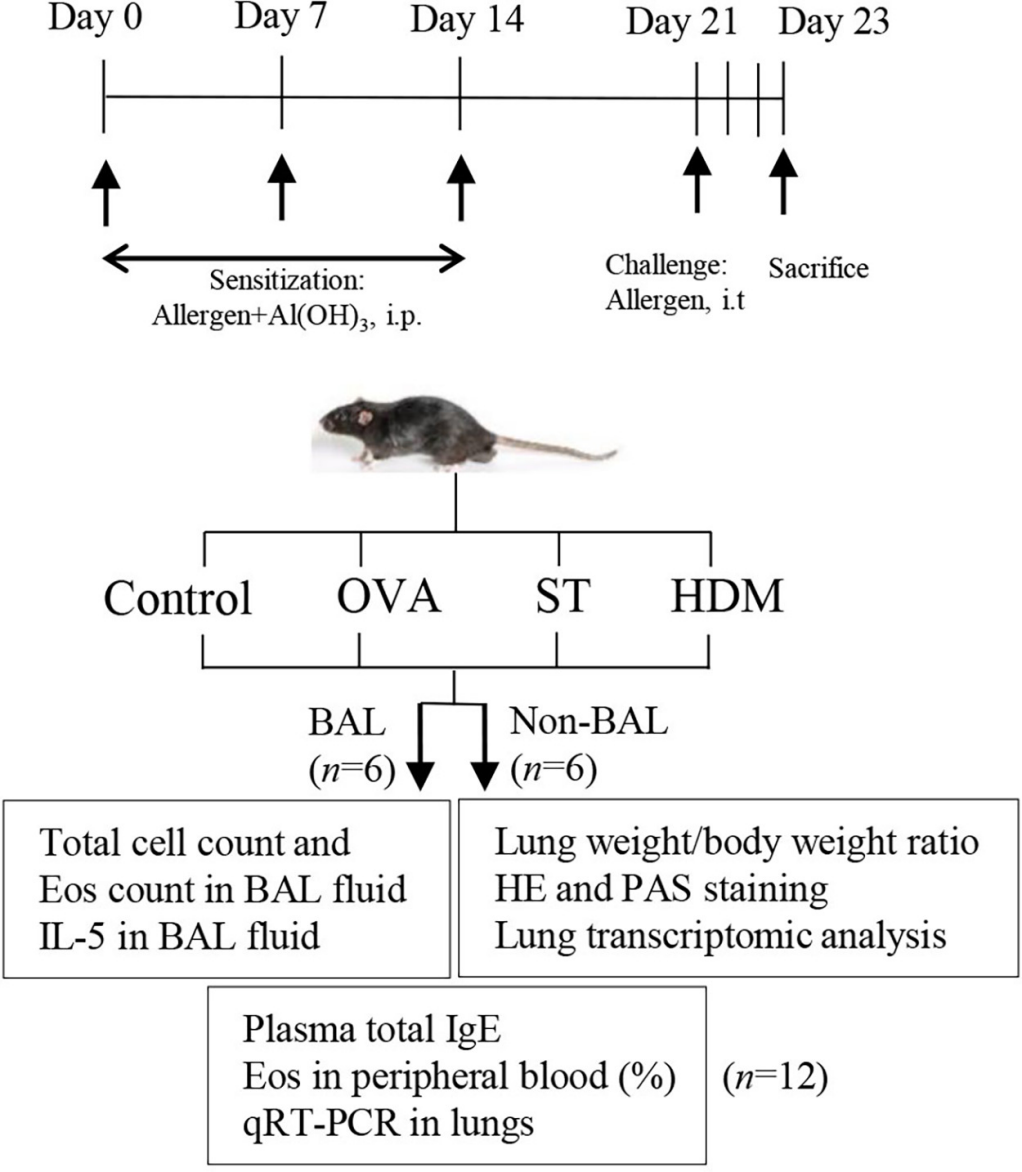
Experimental protocol. Mice were immunized intraperitoneally (i.p.) on days 0, 7, and 14 with 20 μg of allergen (OVA, ST, or HDM) plus alum and were challenged intratracheally (i.t.) with 40 μg of allergen once. On day 23, mice were sacrificed to collect blood, bronchoalveolar lavage (BAL) fluid, and lung tissues for the following analyses: lung weight/body weight ratio, plasma total IgE levels, percentage of eosinophils in peripheral blood, total cell count and eosinophil count in BAL fluid, IL-5 levels in BAL fluid, Hematoxylin-Eosin (HE) staining, Periodic acid–Schiff (PAS) staining, quantitative real-time polymerase chain reaction (qRT-PCR) of lung tissues, and transcriptomic analysis of lung tissues.

A total of 48 female C57BL/6J mice (6–8 weeks, 16–18 g) were obtained from the Comparative Medicine Centre of Yangzhou University. We prepared a 200 μg/ml antigen solution and used the Chromogenic LAL Endotoxin Assay Kit (Beyotime Biotechnology, Haimen, Jiangsu, China) to measure the lipopolysaccharide (LPS) content. The LPS concentrations in the ST, OVA, and HDM solutions were 1.064 EU/ml, 1.375 EU/ml, and 1.541 EU/ml, respectively. Each mouse received an intraperitoneal (i.p.) injection of 20 μg of antigen (in 100 μl). Consequently, the single LPS exposure doses per animal in the ST, OVA, and HDM groups were 0.1064 EU, 0.1375 EU, and 0.1541 EU, respectively. Mice were randomized into four groups: Control, OVA, ST, and HDM (*n* = 12 per group). ST was prepared according to our previous study (13). The OVA, ST, and HDM groups were i.p. sensitized with a suspension containing 20 μg OVA (Grade V, Sigma-Aldrich, St. Louis, MO), ST, or HDM (*Dermatophagoides pteronyssinus*, Greer Labs, USA) mixed with 1.25 mg Al(OH)_3_ on days 0, 7, and 14. On day 21, mice were i.p. anesthetized with 20 ml/kg Avertin (Najing Aibei Biotechnology Co.,Ltd) and challenged with 40 μg OVA, ST, or HDM via direct intratracheal (i.t.) injection. On day 23, six mice from each group were sacrificed to collect blood and lung tissues for the following analyses: lung weight/body weight ratio, plasma total IgE levels, percentage of eosinophils in peripheral blood, Hematoxylin-Eosin (HE) staining, Periodic acid–Schiff (PAS) staining, quantitative real-time polymerase chain reaction (qRT-PCR) of lung tissues, and transcriptomic analysis of lung tissues. Another six mice from each group were used to collect blood, bronchoalveolar lavage (BAL) fluid, and lung tissues for the following analyses: plasma total IgE levels, percentage of eosinophils in peripheral blood, total cell count and eosinophil count in BAL fluid, interleukin (IL)-5 levels in BAL fluid, and qRT-PCR of lung tissues. The animal experimental protocol is illustrated in Figure 1 and was approved by the Animal Ethics Committee of Yangzhou University Medical College (YXYLL-2024-108).

### 2.2 Assessment of plasma IgE and the percentage of eosinophils in peripheral blood

On day 23, mice were anesthetized, and approximately 500 μl of blood was collected via ocular bleeding into heparin-coated tubes. The percentage of eosinophils in peripheral blood was determined using a Mindray BC-5000 Vet hematology analyzer (Shenzhen Mindray Animal Medical Technology Co., Ltd., China). After centrifugation, plasma IgE levels were quantified using an ELISA kit (Elabscience Biotechnology Co., Ltd., Wuhan, China).

### 2.3 Assessment of BAL fluid cellularity and IL-5 in BAL fluid

BAL was performed on six mice from each group using 0.8 ml of sterile saline, as previously described (14). The total cell count was measured using a CellDrop automatic cell counter (DeNovix, USA). BAL fluid was centrifuged using a Cytospin (Thermo Fisher Scientific, USA) at 500 × g for 5 min, and cell smears were prepared for HE staining to observe cell morphology. IL-5 levels in BAL fluid were determined using ELISA kits (BioLegend Co., USA).

### 2.4 Lung weight/body weight ratio and lung transcriptomic analysis

Another six mice from each group were not subjected to BAL. The lungs were dissected, weighed, and the lung weight/body weight ratios were calculated. The left lungs from six mice in the Control, ST, and HDM groups were collected for transcriptome sequencing. Lung tissue samples were sent to Annoroad Gene Tech. Co., Ltd. (Beijing, China). RNA was extracted, and the quality of total RNA samples was assessed. The MGIEasy RNA Library Preparation Kit was used for library construction. After loading the DNA nanoballs onto the chip, sequencing was performed using the DNBSEQ-T7 platform (MGI Tech Co., Ltd., Shenzhen, China). Genes with a fold change (FC) of less than −1.5 or greater than 1.5 were defined as differentially expressed and selected for further analysis. Gene set enrichment analysis (GSEA) of the RNA sequencing data was performed using the C5 ontology gene sets.

### 2.5 Histological observation of lung tissue

Paraffin-embedded lung tissues were sectioned and stained with HE and PAS methods to assess lung inflammation and mucus overproduction in the airways. Images were acquired using an Eclipse 80i microscope (Nikon, Japan). Inflammation was scored as follows: 0 (no inflammation), 1 (a few cells), 2 (a single layer of cells), 3 (2–4 layers of cells), 4 (focal inflammatory cell infiltration), or 5 (intense inflammatory infiltration) on HE-stained lung tissues, as previously described (13).

### 2.6 qRT-PCR

BAL-treated (lavaged) or non-BAL-treated (intact) lung tissues were lysed using TRIzol reagent (Invitrogen, USA) following the manufacturer's protocol. Primer sequences were obtained from PrimerBank (https://pga.mgh.harvard.edu/primerbank/) and are listed in Supplementary Table S1. Specific primers were synthesized by Shanghai Sangon Biotech Co. (Shanghai, China). Reverse transcription and qRT-PCR were performed using the HiScript III RT SuperMix Kit and ChamQ Universal SYBR qPCR Master Mix Kit (Vazyme Biotech Co., Ltd., Nanjing, Jiangsu, China), respectively. The relative expression of genes was analyzed using the 2^−ΔΔCt^ method and normalized to *Gapdh*.

### 2.7 Statistical analysis

Data are expressed as mean ± standard deviation and analyzed using Prism 8.0 GraphPad Software. Data normality was assessed using the Shapiro–Wilk test, while homogeneity of variances was verified through Bartlett's test. For datasets satisfying both normality and equal variance assumptions, statistical differences were determined by one-way ANOVA followed by Tukey's multiple comparisons test. Non-normally distributed data or datasets with heterogeneous variances were analyzed using the Kruskal–Wallis nonparametric test followed by Dunn's multiple comparisons test. For lung inflammation scores and *Ccl-24* mRNA level, nonparametric tests (Kruskal–Wallis test and Dunn's test) were used, and data are expressed as median ± range. Differences were considered statistically significant at *P* < 0.05.

## 3 Results

### 3.1 ST and HDM increase lung-to-body weight ratios and the percentage of eosinophils in peripheral blood

**Figure 2:**
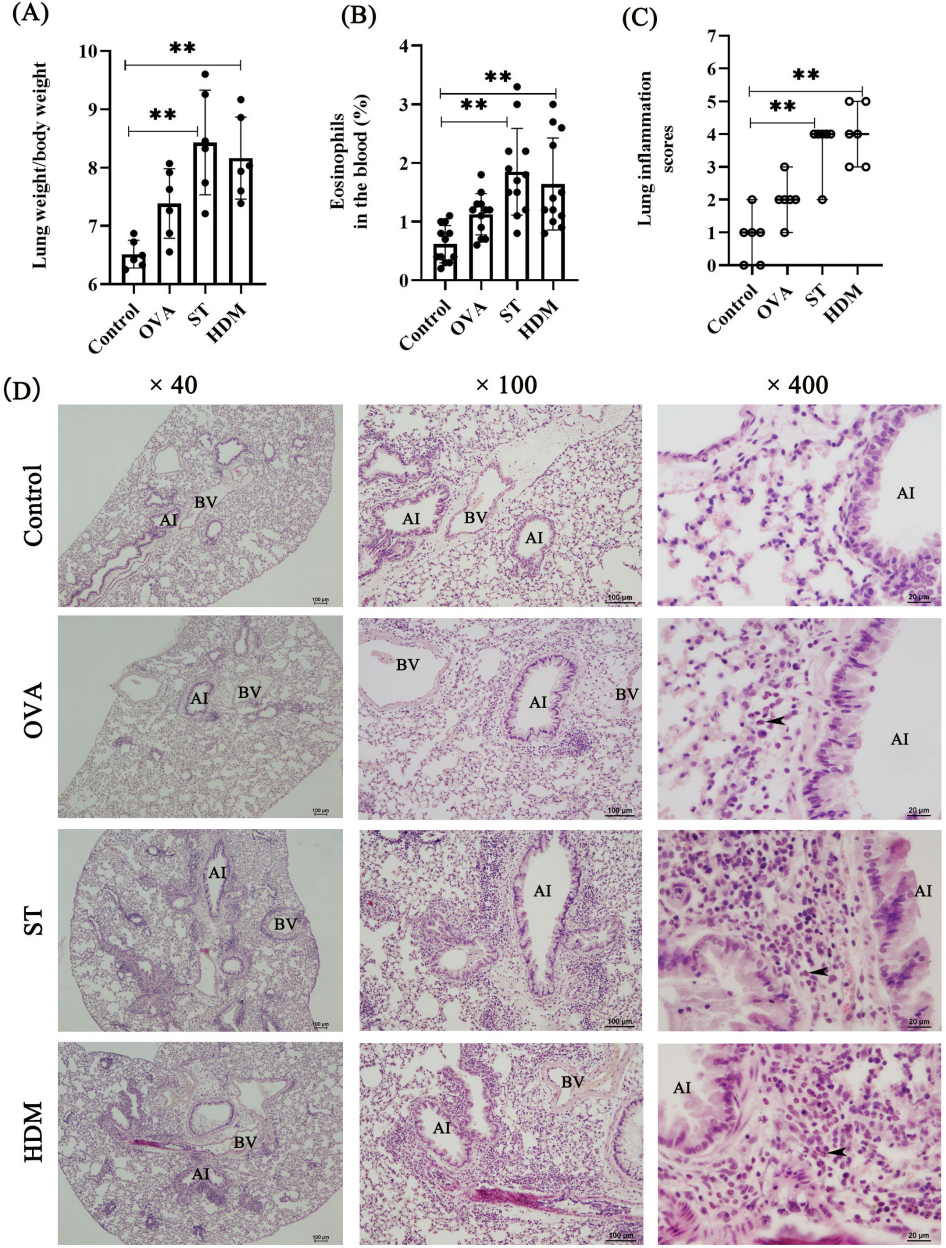
ST and HDM induce airway inflammation. **(A)** Measurement of the lung weight/body weight ratio (*n* = 6). **(B)** Percentage of eosinophils in peripheral blood (*n* = 12). **(C)** Lung inflammation scores (*n* = 6); data represent median ± range. **(D)** HE staining (4×, scale bar = 100 µm; 100×, scale bar = 100 µm; 400×, scale bar = 20 µm). Arrows indicate eosinophils. AI, airway; BV, blood vessel. Data represent mean ± SD. *P < 0.05, **P < 0.01.

Eosinophil activation and pulmonary infiltration are hallmark features of allergic asthma. To evaluate allergen-induced pulmonary inflammation, we assessed the lung-to-body weight ratio. Mice immunized with ST and HDM exhibited a significantly higher lung-to-body weight ratio compared to the control group (Figure 2A). Asthma patients exhibiting elevated blood eosinophil levels tend to have a higher frequency of asthma attacks compared to those with lower eosinophil counts (15). As showed in the Figure 2B, ST- and HDM-treated mice showed a marked increase in the percentage of eosinophils in peripheral blood, suggesting systemic eosinophil activation.

### 3.2 ST and HDM induce eosinophilic inflammation in airways, elevate the total cell count and eosinophil count in the BAL fluid

Histopathological analysis of HE-stained lung tissues revealed distinct airway inflammatory responses. Control mice displayed normal lung morphology, while ST- and HDM-treated mice exhibited pronounced eosinophil accumulation around bronchi and blood vessels. In contrast, a single dose of OVA induced only mild airway inflammation, indicating that repeated OVA exposures may be necessary to elicit a robust inflammatory response. Consistent with these findings, the lung inflammation scores were significantly elevated in the lung tissues of ST- and HDM-treated mice compared to the control and OVA groups (Figures 2C,D).

**Figure 3:**
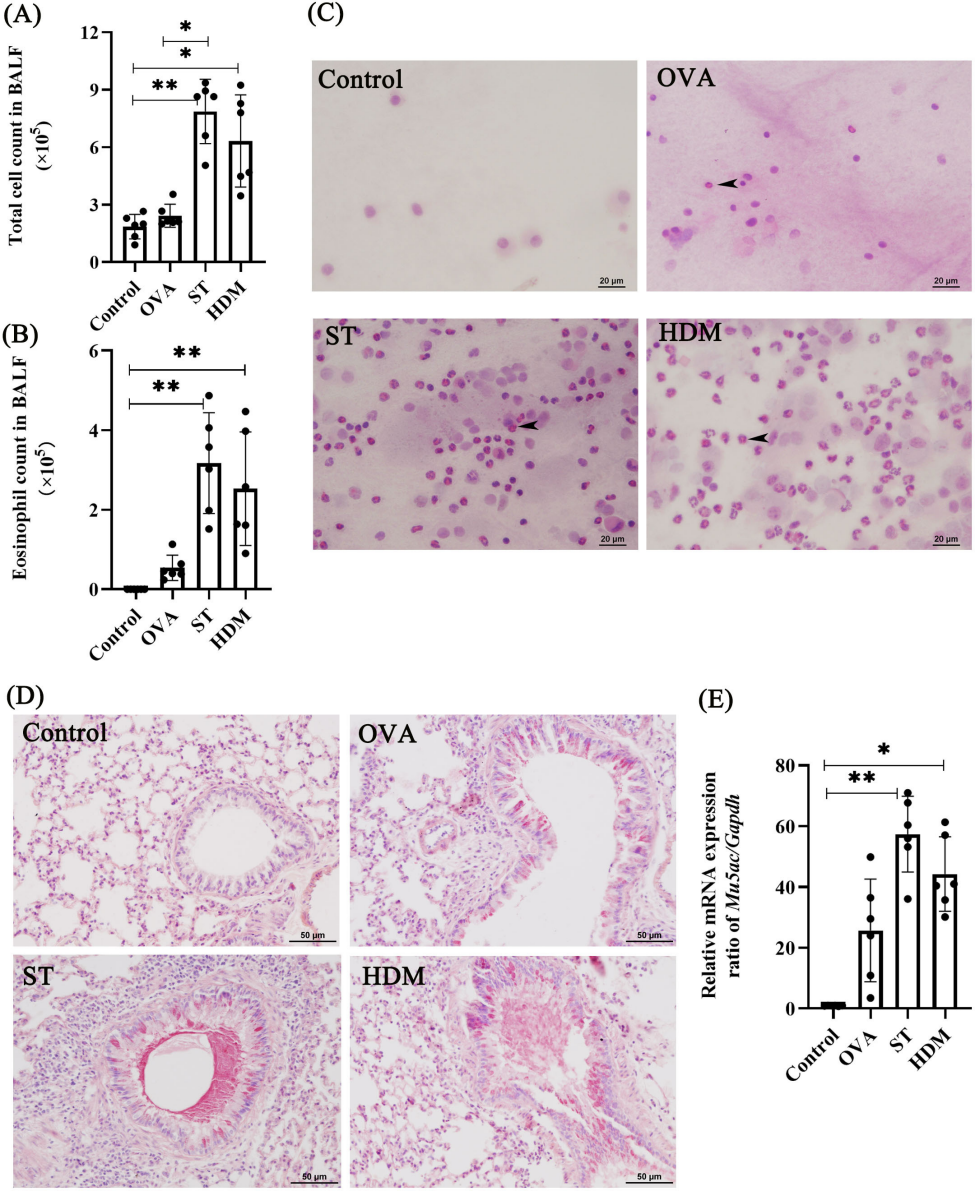
ST and HDM induce increased the eosinophil count in BAL fluid and mucus hypersecretion in the lung tissues. **(A)** Total cell count in BAL fluid (*n* = 6). **(B)** Eosinophil count in BAL fluid (*n* = 6). **(C)** BAL fluid cellularity (HE staining, 400×, scale bar = 20 µm). Arrows indicate eosinophils. **(D)** PAS staining (200×, scale bar = 50 µm). **(E)** *Muc5ac* mRNA levels in lung tissues (*n* = 6). Data represent mean ± SD. *P < 0.05, **P < 0.01.

BAL fluid analysis further corroborated these results. Macrophages were the predominant cell type in BAL fluid from the control group. A few eosinophils were detected in the OVA group, and ST and HDM treatment significantly increased total cell counts in BAL fluid, with a notable influx of eosinophils (identified by characteristic red cytoplasmic staining). Additionally, neutrophils were observed in the HDM group (Figures 3A–C). These findings collectively demonstrate that ST and HDM enhance eosinophil-driven airway inflammation.

### 3.3 ST and HDM trigger mucus hypersecretion and induce Muc5ac upregulation

Mucus hypersecretion is considered a key pathological characteristic of asthma, often linked to extensive mucus plugging in the airways (16). There was a significant positive correlation between sputum MUC5AC levels and the proportion of eosinophils in steroid-untreated patients with mild asthma (17). PAS staining revealed significant mucus hypersecretion in the airways of ST- and HDM-treated mice, with positive signals detected in both goblet cells and airway lumens. In contrast, OVA treatment resulted in milder mucus production in goblet cells. Consistent with these observations, the expression of *Muc5ac*, a major airway mucin, was significantly upregulated in all experimental groups, with the highest levels observed in ST- and HDM-treated mice (Figures 3D,E).

### 3.4 ST and HDM elevate plasma IgE and Th2 cytokines

**Figure 4:**
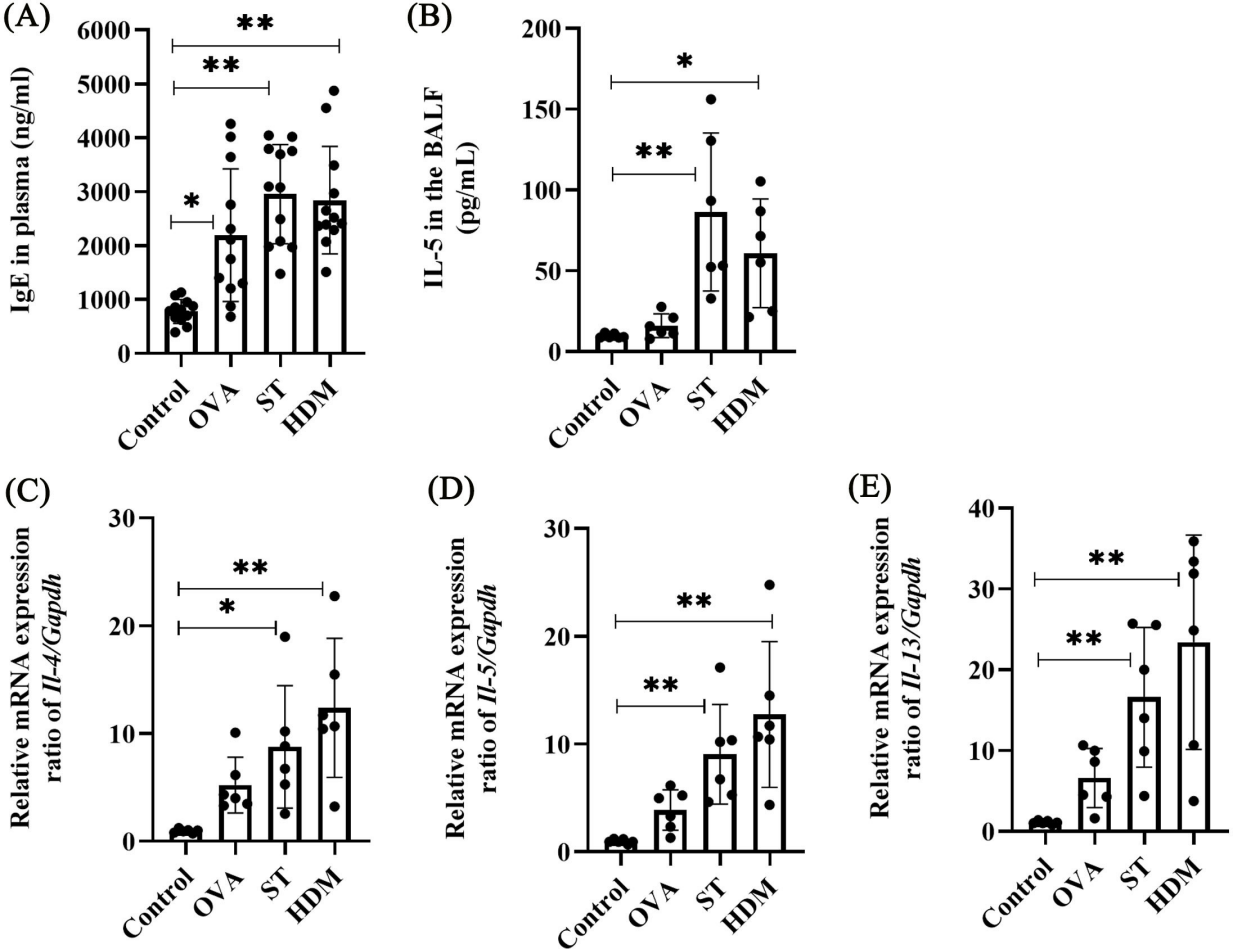
ST and HDM elevate plasma IgE and Th2 cytokines. **(A)** Plasma IgE levels (*n* = 12). **(B)** IL-5 levels in BAL fluid (*n* = 6). **(C)** *Il-4* mRNA levels in lung tissues (*n* = 6). **(D)** *Il-5* mRNA levels in lung tissues (*n* = 6). **(E)** *Il-13* mRNA levels in lung tissues (*n* = 6). Data represent mean ± SD. *P < 0.05, **P < 0.01.

IgE, a central mediator of allergic responses, was significantly elevated in the plasma of OVA-, ST-, and HDM-treated mice compared to controls (Figure 4A). IL-4 and IL-13, critical cytokines for IgE production and Th2 polarization, were also assessed. While a single OVA challenge did not significantly increase IL-5 levels in BAL fluid or the mRNA expression of *Il-4*, *Il-5*, and *Il-13* in lung tissues, ST and HDM treatment markedly upregulated these Th2 cytokines (Figures 4B–E, Supplementary Figures S1A–C). These results highlight the potent Th2-skewing capacity of ST and HDM.

### 3.5 RNA sequencing identifies differential gene expression in HDM- and ST-treated lungs

**Figure 5:**
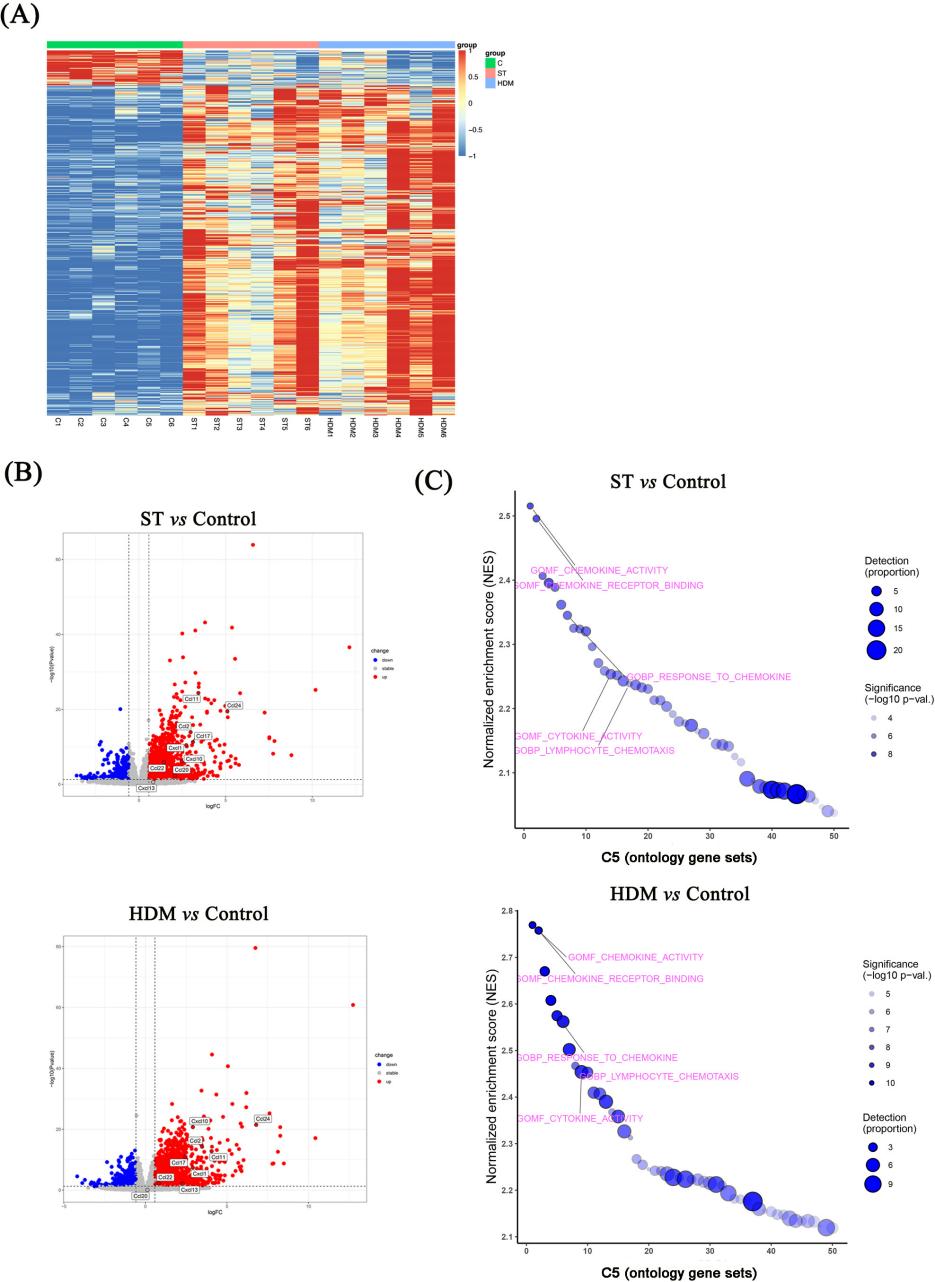
RNA sequencing of lung tissues from ST- and HDM-treated mice. **(A)** Heatmaps showing differentially expressed genes (DEGs) in ST- and HDM-treated mice compared to control mice (*n* = 6). **(B)** Volcano plots showing DEGs between ST- or HDM-treated mice and control mice (*n* = 6). **(C)** Gene set enrichment analysis (GSEA) of RNA sequencing data.

Given the relatively weak inflammatory and Th2 responses induced by OVA, we only performed RNA sequencing on lung tissues from control, ST-, and HDM-treated mice. The results, visualized using heatmaps and volcano plots (Figures 5A,B), revealed 2,563 differentially expressed genes (DEGs) in HDM-treated mice (856 downregulated and 1,707 upregulated) and 1,952 DEGs in ST-treated mice (573 downregulated and 1,379 upregulated) (FC ≥1.5, *P* < 0.05).

**Figure 6:**
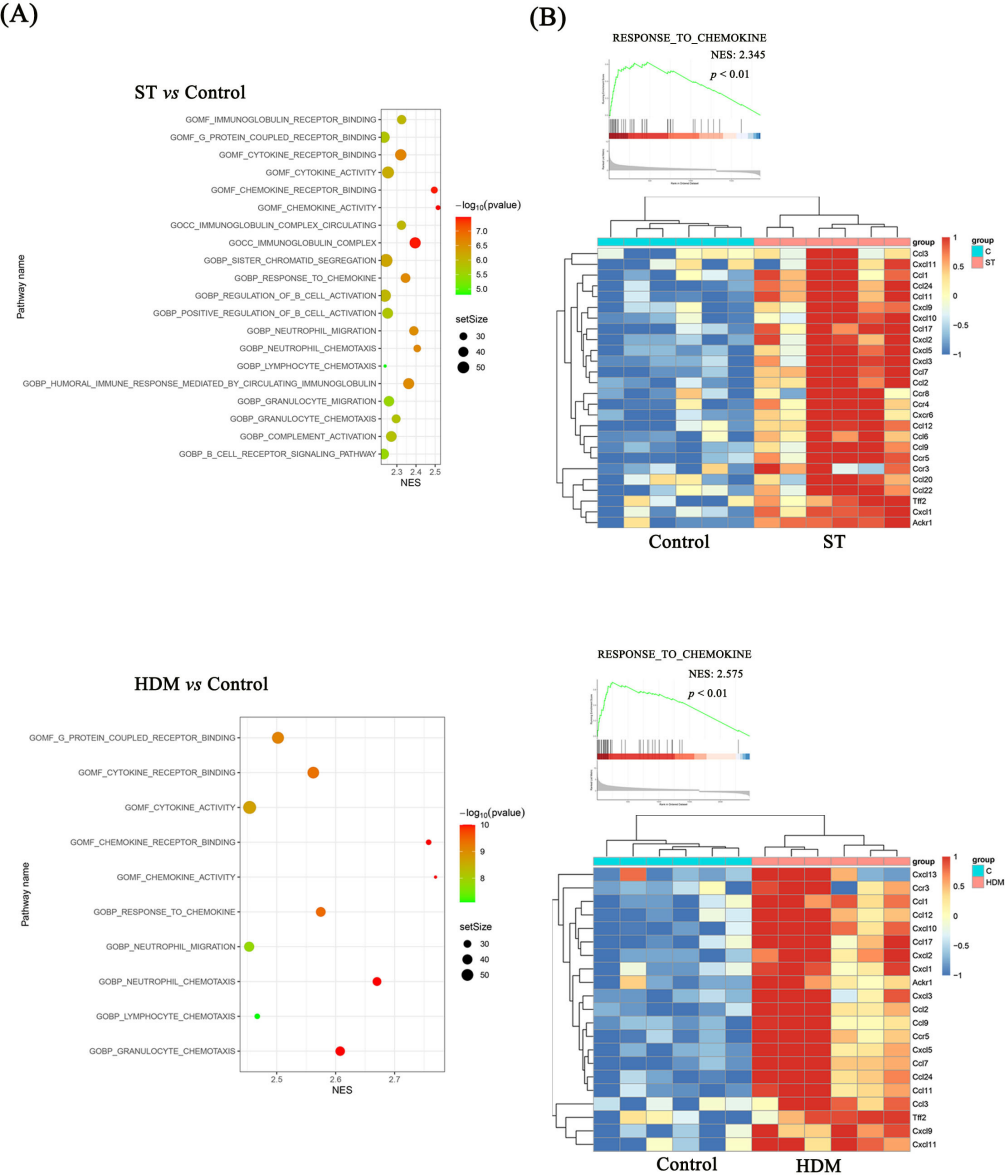
GSEA analysis of RNA sequencing data. **(A)** GSEA analysis showing the top 20 pathways for Control vs. ST and the top 10 pathways for Control vs. HDM. **(B)** DEGs in the “Response to chemokine” pathway.

Subsequent GSEA using the C5 ontology gene sets highlighted key pathways associated with the observed DEGs. In the ST group, GSEA analysis of DEGs indicated significant enrichment in chemokine activity, chemokine receptor binding, response to chemokines, neutrophil chemotaxis, and neutrophil migration, based on the top 10 normalized enrichment scores (NES). Similarly, in HDM-treated mice, enriched pathways included chemokine activity, chemokine receptor binding, response to chemokines, neutrophil chemotaxis, cytokine receptor binding, and cytokine activity (Figures 5C, 6A).

These findings suggest that ST and HDM induce pulmonary inflammation through distinct but overlapping chemokine-related pathways, with a prominent role for neutrophil and cytokine-mediated mechanisms.

### 3.6 ST and HDM upregulate Cxcl-1, Cxcl-13, Ccl-2, Ccl-11, Ccl-17, and Ccl-24

**Figure 7:**
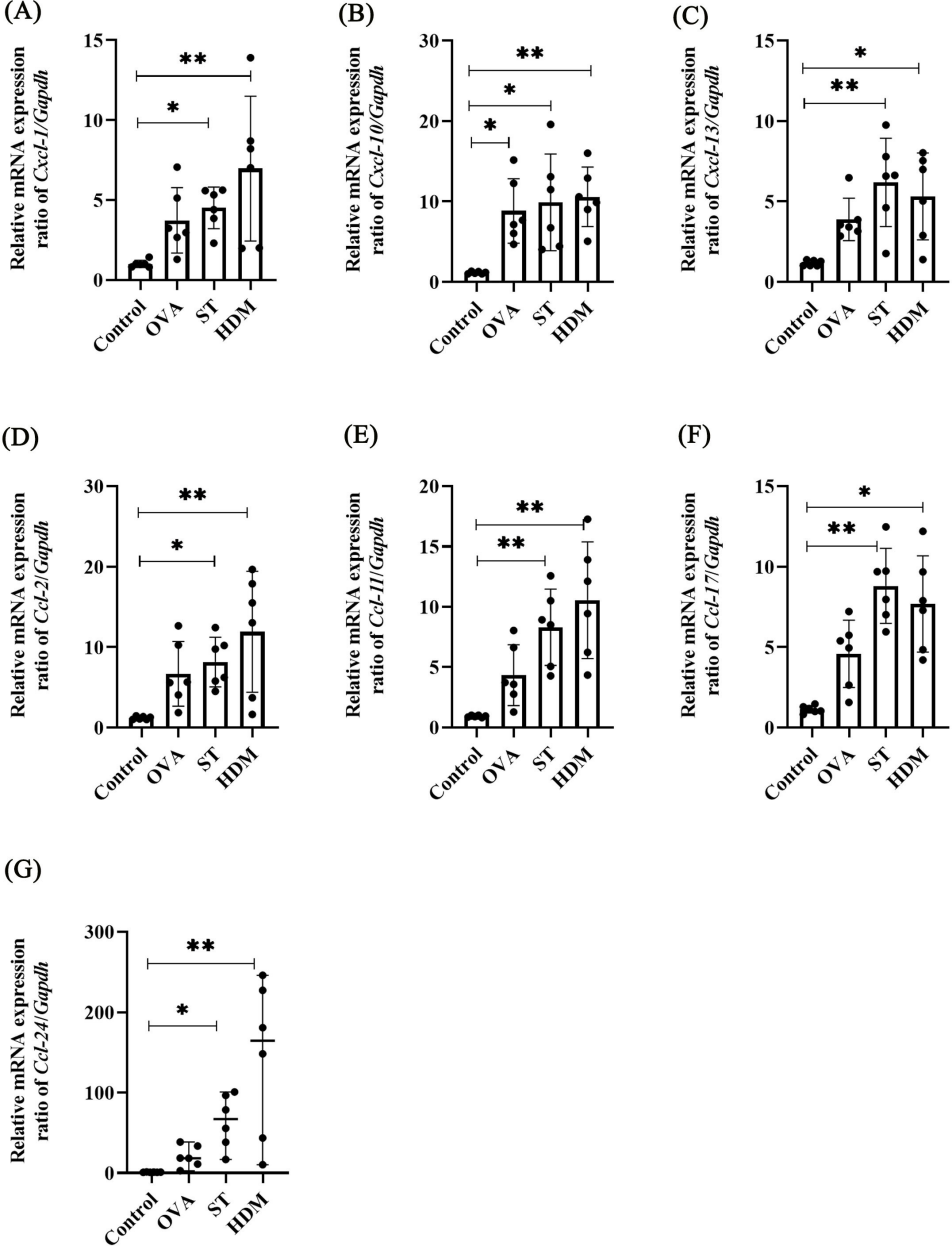
Chemokine levels in OVA-, ST-, and HDM-treated mice. mRNA levels of *Cxcl1* **(A)**, *Cxcl10* **(B)**, *Cxcl13* **(C)**, *Ccl2* **(D)**, *Ccl11* **(E)**, *Ccl17* **(F)**, and *Ccl24* **(G)** in the non-BAL-treated (intact) lung tissues (*n* = 6). Data represent mean ± SD. For *Ccl-24* mRNA level, data are expressed as median ± range. *P < 0.05, **P < 0.01.

As shown in Figure 6B, the expression levels of chemokine-related genes, including *Ccl-2*, *Ccl-11*, *Ccl-17*, *Ccl-24*, *Cxcl-1*, *Cxcl-10*, were significantly higher in both ST- and HDM-treated mice compared to the control group. These findings were further validated by qRT-PCR analysis of mRNA extracted from lung tissues. In the Figure 7 and Supplementary Figures S1D–J, a single exposure of ST or HDM commonly upregulated the expression of *Cxcl-1* [neutrophil recruitment (18)], *Cxcl-13* [B cell recruitment (19)], *Ccl-2* [monocyte infiltration (20)], *Ccl-11*, and *Ccl-24* [eosinophil recruitment (21)], and *Ccl-17* [Th2 polarization (22)]. Additionally, *Cxcl-10* [a marker of Th1 activation (23)] was upregulated across all three allergen groups (ST, HDM, and OVA). Quantitative analysis of inflammatory markers and chemokine profiles demonstrates that ST and HDM trigger immune cell recruitment in this acute challenge model.

## 4 Discussion

In murine models of acute asthma, OVA is typically utilized to induce systemic sensitization through intraperitoneal administration over 2–3 weeks with aluminum hydroxide adjuvant, followed by airway challenges via aerosolized OVA inhalation (1%–5%) or intranasal OVA instillation for 3–7 days. In contrast, HDM sensitization does not require adjuvants, and the protocol duration is shorter (7–14 days) (3). Notably, varying doses of HDM can elicit distinct asthma phenotypes, including eosinophilic, mixed, and neutrophilic inflammation (24). However, to establish a neutrophil-predominant asthma model using OVA, concurrent administration of complete Freund's adjuvant (CFA) and LPS is required (25). Our previous studies demonstrated that ST i.n. challenge induces significantly stronger airway inflammation and Th2-polarized immune responses compared to OVA, irrespective of adjuvant use. Although intratracheal instillation is an invasive technique (generally administered as a single dose), it enables the accurate and controlled delivery of an antigen quantity to the lungs. This precision is particularly advantageous when comparing the potency of different allergens. In this study, the protocol was modified that mice were sensitized via three i.p. injections of OVA, HDM, or ST mixed with alum adjuvant, followed by a single intratracheal challenge with the respective allergen. Results showed that the plasma IgE was elevated in all allergen-immunized mice, indicating enough systemic sensitization via three i.p. injections of allergens. However, both ST and HDM challenge increased lung-to-body weight ratio, airway eosinophil infiltration, the total cell count and eosinophil count in the BAL fluid, after only once i.t. challenge, compared to those in OVA-treated mice.

The potent inflammatory effects of HDM are largely attributed to Der p 1, a major HDM allergen. Der p 1 disrupts epithelial tight junctions (ZO-1 or occludin) (26) and activates protease-activated receptor 2 (PAR2) (5), directly triggering the release of alarmins such as TSLP and IL-33 from epithelial cells. These alarmins play a pivotal role in driving the upregulation of Ccl-17 and Ccl-22 (27), linking HDM exposure to Th2 polarization. Additionally, HDM stimulates group 2 innate lymphoid cells, leading to the production of IL-5 and IL-13, which further amplify eosinophilic inflammation and Th2 responses (28). Beyond epithelial cells, HDM also activates alveolar macrophages through the TLR4/CD14 receptor complex, initiating pro-inflammatory signaling pathways (6). HDM exploits TLR2 as a central hub to drive nasal inflammation. β-glucans in HDM activate TLR2 in nasal epithelial cells to promote allergic rhinitis via DUOX2/ROS-mediated signaling (29). HDM allergens also engage TLR2 in dendritic cells to upregulate c-kit and costimulatory molecules (CD80/CD86), thereby polarizing Th2 responses and exacerbating asthma (30). Unlike HDM which activates both TLR2 and TLR4 pathways, OVA lacks intrinsic TLR-stimulating capacity and requires re-exposure to prime immune responses. This fundamental difference likely explains the weaker efficacy of OVA in single-exposure models compared to HDM, which can robustly induce allergic inflammation through multiple synergistic mechanisms.

In this study, ST or HDM challenge significantly upregulated the mRNA levels of chemokines *Ccl-11*, *Ccl-24*, *Ccl-17*, *Ccl-2*, and *Cxcl-1*, which are associated with eosinophil recruitment, Th2 cell activation, monocyte infiltration and neutrophil activation. *Cxcl-13* was upregulated in the lung tissues of ST- or HDM-treated mice. As a chemokine essential for germinal center formation and B cell polarization (31), CXCL-13 may also contribute to local IgE production in asthma. This chemokine synergy likely contributes to the enhanced severity of ST- or HDM-induced asthma compared to the OVA model. Notably, HDM induces dual pro-inflammatory mechanisms: airway epithelial cells produce CCL-17, and *in vitro* studies demonstrate that HDM exposure primes macrophages to adopt an M2-like phenotype with elevated CCL-17 production (32). Furthermore, human CD11c^+^ dendritic cells derived from HDM-allergic patients exhibited HDM-triggered CCL-17 release (33), initiating a chemotactic cascade that recruits polarized Th2 cells and subsequently promotes eosinophil infiltration. The neutrophilic component of this inflammation is particularly clinically relevant. Neutrophils, traditionally associated with steroid-resistant asthma, demonstrate a positive correlation with asthma severity (34). HDM enhances secretion of neutrophil survival factors (IL-6, IL-8, CCL-2, GM-CSF) in both physiological and allergic conditions and prolong neutrophil survival via PAR2-mediated apoptosis inhibition (35). Critically, the co-expression of Th2-polarizing CCL-17 and neutrophil-recruiting CXCL-1 establishes a feedforward loop wherein Th2 cytokines further amplify chemokine production, deteriorating airway inflammation.

Additionally, OVA, ST or HDM induced shared upregulation of CXCL-10 (a Th1-associated chemokine). Early Th1 activation, marked by CXCL-10 upregulation, may counteract type 2 immune responses. This is supported by studies showing that low-dose LPS exposure attenuates OVA-induced airway inflammation through enhanced Th1 cytokine production and suppressed Th2 cytokine release (36). The C57BL/6 strain, which is prone to a mixed Th1/Th2 phenotype, exhibited elevated levels of CCL-11 and CCL-5 in the BAL fluid of OVA-induced mice (37). Additionally, the antigen solution used in this study contains LPS, a factor that could influence inflammatory outcomes. While LPS contamination likely contributes to Cxcl-10 upregulation across all allergens, the ST/HDM-specific induction of Ccl-2 and Cxcl-1 implies synergistic effects between LPS and their intrinsic components (e.g., proteases, β-glucans), which may activate epithelial-innate immune crosstalk to amplify neutrophilic/monocytic inflammation. These findings imply that Th1 activation could vary depending on the mouse strain or LPS exposure levels.

Most ST studies focus on oral sensitization. ST's allergenicity is critically dependent on its conformational epitopes. Five dominant IgE-binding regions have been identified in Pen a 1: residues 43–57, 85–105, 133–148, 187–202, and 247–284 (38). These conformational epitopes are highly sensitive to structural perturbations: thermal processing partially unfolds ST, exposing linear epitopes that maintain systemic anaphylaxis in mice comparable to raw ST, while reverse-pressure sterilization induces protein aggregation, masking heat/digestion-stable epitopes (e.g., arginine kinase Glu59-Ser63; sarcoplasmic calcium-binding protein Asn57-Phe67) and significantly reducing IgE binding (39). Crucially, airway exposure to ST circumvents gastrointestinal processing, allowing intact conformational epitopes to directly engage lung dendritic cells—a mechanism implicated in occupational shrimp allergy, where aerosolized ST particles correlate with elevated respiratory symptoms and asthma biomarkers in shrimp processing workers (40). While our study demonstrates ST's capacity to induce eosinophilic inflammation comparable to HDM, key mechanistic differences remain unexplored. Unlike HDM that directly disrupt epithelial barriers via PAR2 activation, ST may employ alternative pathways for immune priming.

## 5 Conclusion

These findings demonstrate that ST is a potent allergen for modeling allergic asthma, characterized by robust eosinophilic inflammation and elevated type 2 cytokines/chemokines, closely resembling the effects of HDM.

## Data Availability

The original contributions presented in the study are included in the article/Supplementary Material, further inquiries can be directed to the corresponding author.

## Supplementary material

Supplementary Figure S1 Th2 cytokines and chemokine levels in OVA-, ST-, and HDM-treated mice. mRNA levels of *Il-4* **(A)**, *Il-5* **(B)**, Il-13 **(C)**, *Cxcl1* **(D)**, *Cxcl10* **(E)**, *Cxcl13* **(F)**, *Ccl2* **(G)**, *Ccl11* **(H)**, *Ccl17* **(I)**, and *Ccl24* **(J)** in the BAL-treated (lavaged) lung tissues (*n* = 6). Data represent mean ± SD. For *Ccl-24* mRNA level, data are expressed as median ± range. **P* < 0.05, ***P* < 0.01.

## References

[1] Jeebhay MF Baatjies R. Occupational inhalant allergy in food handling occupations. Curr Opin Allergy Clin Immunol. (2022) 22:64–72. 10.1097/ACI.0000000000000804 34923552

[2] Akar-Ghibril N Casale T Custovic A Phipatanakul W. Allergic endotypes and phenotypes of asthma. J Allergy Clin Immunol Pract. (2020) 8:429–40. 10.1016/j.jaip.2019.11.008 32037107 PMC7569362

[3] Feng Y Xu L Zhang J Bin J Pang X He S Allergenic protein-induced type I hypersensitivity models: a review. Front Allergy. (2024) 5:1481011. 10.3389/falgy.2024.1481011 39483683 PMC11525013

[4] Yao L Yuan X Fu H Guo Q Wu Y Xuan S Epithelium-derived cystatin SN inhibits house dust mite protease activity in allergic asthma. Allergy. (2023) 78:1507–23. 10.1111/all.15739 37026502

[5] Kato T Takai T Fujimura T Matsuoka H Ogawa T Murayama K Mite serine protease activates protease-activated receptor-2 and induces cytokine release in human keratinocytes. Allergy. (2009) 64:1366–74. 10.1111/j.1398-9995.2009.02023.x 19416145

[6] Liu CF Drocourt D Puzo G Wang JY Riviere M. Innate immune response of alveolar macrophage to house dust mite allergen is mediated through TLR2/-4 co-activation. PLoS One. (2013) 8:e75983. 10.1371/journal.pone.0075983 24098413 PMC3787959

[7] Spolidoro GCI Ali MM Amera YT Nyassi S Lisik D Ioannidou A Prevalence estimates of eight big food allergies in Europe: updated systematic review and meta-analysis. Allergy. (2023) 78:2361–417. 10.1111/all.15801 37405695

[8] Asero R Pravettoni V Scala E Villalta D. House dust mite-shrimp allergen interrelationships. Curr Allergy Asthma Rep. (2020) 20:9. 10.1007/s11882-020-0902-2 32144500

[9] Lopez-Matas MA Iraola V Moya R Vailes LD Pomes A Boquete M Cloning and characterization of tropomyosin from the mite chortoglyphus arcuatus. Mol Immunol. (2015) 68:634–40. 10.1016/j.molimm.2015.10.004 26522591

[10] Wong L Huang CH Lee BW. Shellfish and house dust mite allergies: is the link tropomyosin? Allergy Asthma Immunol Res. (2016) 8:101–6. 10.4168/aair.2016.8.2.101 26739402 PMC4713872

[11] Farioli L Losappio LM Giuffrida MG Pravettoni V Micarelli G Nichelatti M Mite-induced asthma and IgE levels to shrimp, mite, tropomyosin, arginine kinase, and der p 10 are the most relevant risk factors for challenge-proven shrimp allergy. Int Arch Allergy Immunol. (2017) 174:133–43. 10.1159/000481985 29169170

[12] Fang L Yan Y Xu Z He Z Zhou S Jiang X Tectochrysin ameliorates murine allergic airway inflammation by suppressing Th2 response and oxidative stress. Eur J Pharmacol. (2021) 902:174100. 10.1016/j.ejphar.2021.174100 33878335

[13] Fang L Zhou F Wu F Yan Y He Z Yuan X A mouse allergic asthma model induced by shrimp tropomyosin. Int Immunopharmacol. (2021) 91:107289. 10.1016/j.intimp.2020.107289 33370683

[14] Li X Hou R Ding H Gao X Wei Z Qi T Mollugin ameliorates murine allergic airway inflammation by inhibiting Th2 response and M2 macrophage activation. Eur J Pharmacol. (2023) 946:175630. 10.1016/j.ejphar.2023.175630 36871665

[15] Tran TN Khatry DB Ke X Ward CK Gossage D. High blood eosinophil count is associated with more frequent asthma attacks in asthma patients. Ann Allergy Asthma Immunol. (2014) 113:19–24. 10.1016/j.anai.2014.04.011 24846699

[16] Wang X Yang X Li Y Wang X Zhang Y Dai X Lyn kinase represses mucus hypersecretion by regulating IL-13-induced endoplasmic reticulum stress in asthma. EBioMedicine. (2017) 15:137–49. 10.1016/j.ebiom.2016.12.010 28024734 PMC5233819

[17] Tajiri T Matsumoto H Jinnai M Kanemitsu Y Nagasaki T Iwata T Pathophysiological relevance of sputum MUC5AC and MUC5B levels in patients with mild asthma. Allergol Int. (2022) 71:193–9. 10.1016/j.alit.2021.09.003 34656442

[18] Yang D Li Y Liu T Yang L He L Huang T IL-1beta promotes IL-17A production of ILC3s to aggravate neutrophilic airway inflammation in mice. Immunology. (2023). 10.1111/imm.13644 36988516

[19] Ishihara S Shoda T Ishimura N Ohta S Ono J Azuma Y Serum biomarkers for the diagnosis of eosinophilic esophagitis and eosinophilic gastroenteritis. Intern Med. (2017) 56:2819–25. 10.2169/internalmedicine.8763-16 28943560 PMC5709622

[20] Galvan-Blasco P Gil-Serrano J Sala-Cunill A. New biomarkers in anaphylaxis (beyond tryptase). Curr Treat Options Allergy. (2022) 9:303–22. 10.1007/s40521-022-00326-1 36467524 PMC9702867

[21] White JR Lee JM Dede K Imburgia CS Jurewicz AJ Chan G Identification of potent, selective non-peptide CC chemokine receptor-3 antagonist that inhibits eotaxin-, eotaxin-2-, and monocyte chemotactic protein-4-induced eosinophil migration. J Biol Chem. (2000) 275:36626–31. 10.1074/jbc.M006613200 10969084

[22] Pilette C Francis JN Till SJ Durham SR. CCR4 ligands are up-regulated in the airways of atopic asthmatics after segmental allergen challenge. Eur Respir J. (2004) 23:876–84. 10.1183/09031936.04.00102504 15219001

[23] Lam KP Chu YT Kuo CH Wang WL Tok TS Chin YY Suppressive effects of procaterol on expression of IP-10/CXCL 10 and RANTES/CCL 5 by bronchial epithelial cells. Inflammation. (2011) 34:238–46. 10.1007/s10753-010-9229-9 20652827

[24] Tan HT Hagner S Ruchti F Radzikowska U Tan G Altunbulakli C Tight junction, mucin, and inflammasome-related molecules are differentially expressed in eosinophilic, mixed, and neutrophilic experimental asthma in mice. Allergy. (2019) 74:294–307. 10.1111/all.13619 30267575

[25] Xia M Xu F Ni H Wang Q Zhang R Lou Y Neutrophil activation and NETosis are the predominant drivers of airway inflammation in an OVA/CFA/LPS induced murine model. Respir Res. (2022) 23:289. 10.1186/s12931-022-02209-0 36271366 PMC9587569

[26] Wan H Winton HL Soeller C Tovey ER Gruenert DC Thompson PJ Der p 1 facilitates transepithelial allergen delivery by disruption of tight junctions. J Clin Invest. (1999) 104:123–33. 10.1172/JCI5844 10393706 PMC408401

[27] Sato M Matsuo K Susami Y Yamashita A Hayasaka H Hara Y A CCR4 antagonist attenuates atopic dermatitis-like skin inflammation by inhibiting the recruitment and expansion of Th2 cells and Th17 cells. Int Immunol. (2023) 35:437–46. 10.1093/intimm/dxad019 37279584

[28] Li Y Qu Z Wang X Wang Q Lv Z Wang W House dust mite allergen directly activates ILC2 cells via the TLR4 signaling pathway in allergic airway diseases. Cell Immunol. (2024) 405–406:104884. 10.1016/j.cellimm.2024.104884 39437527

[29] Ryu JH Yoo JY Kim MJ Hwang SG Ahn KC Ryu JC Distinct TLR-mediated pathways regulate house dust mite-induced allergic disease in the upper and lower airways. J Allergy Clin Immunol. (2013) 131:549–61. 10.1016/j.jaci.2012.07.050 23036747

[30] Wu W Wang CX Chen H Zhou J Zhang JZ Gao L House dust mite allergens mediate the activation of c-kit in dendritic cells via toll-like receptor 2. Mol Med Rep. (2015) 12:5307–13. 10.3892/mmr.2015.4092 26238189

[31] Guo J Qian J Zhang R. The pathological features of ectopic lymphoid neogenesis in idiopathic dacryoadenitis. BMC Ophthalmol. (2016) 16:66. 10.1186/s12886-016-0250-0 27230507 PMC4882794

[32] Lechner A Henkel FDR Hartung F Bohnacker S Alessandrini F Gubernatorova EO Macrophages acquire a TNF-dependent inflammatory memory in allergic asthma. J Allergy Clin Immunol. (2022) 149:2078–90. 10.1016/j.jaci.2021.11.026 34974067

[33] Perros F Hoogsteden HC Coyle AJ Lambrecht BN Hammad H. Blockade of CCR4 in a humanized model of asthma reveals a critical role for DC-derived CCL17 and CCL22 in attracting Th2 cells and inducing airway inflammation. Allergy. (2009) 64:995–1002. 10.1111/j.1398-9995.2009.02095.x 19630858

[34] Andersson CK Adams A Nagakumar P Bossley C Gupta A De Vries D Intraepithelial neutrophils in pediatric severe asthma are associated with better lung function. J Allergy Clin Immunol. (2017) 139:1819–29.e1811. 10.1016/j.jaci.2016.09.022 27746241 PMC5457125

[35] Lee NR Baek SY Gu A Kim DH Kim SY Lee JS House dust mite allergen suppresses neutrophil apoptosis by cytokine release via PAR2 in normal and allergic lymphocytes. Immunol Res. (2016) 64:123–32. 10.1007/s12026-015-8730-5 26666432

[36] Ding F Fu Z Liu B. Lipopolysaccharide exposure alleviates asthma in mice by regulating Th1/Th2 and treg/Th17 balance. Med Sci Monit. (2018) 24:3220–9. 10.12659/MSM.905202 29768397 PMC5985709

[37] Gueders MM Paulissen G Crahay C Quesada-Calvo F Hacha J Van Hove C Mouse models of asthma: a comparison between C57BL/6 and BALB/c strains regarding bronchial responsiveness, inflammation, and cytokine production. Inflamm Res. (2009) 58:845–54. 10.1007/s00011-009-0054-2 19506803

[38] Ayuso R Lehrer SB Reese G. Identification of continuous, allergenic regions of the major shrimp allergen pen a 1 (tropomyosin). Int Arch Allergy Immunol. (2002) 127:27–37. 10.1159/000048166 11893851

[39] Liu K Lin S Gao X Wang S Liu Y Liu Q Reduced allergenicity of shrimp (*Penaeus vannamei*) by altering the protein fold, digestion susceptibility, and allergen epitopes. J Agric Food Chem. (2023) 71:9120–34. 10.1021/acs.jafc.3c01557 37257052

[40] Zegeye FD Graff P Grgic M Mollerup S Afanou AK Bang BE Respiratory symptoms, sensitisation and occupational exposure in the shrimp processing industry. Front Allergy. (2025) 6:1520576. 10.3389/falgy.2025.1520576 40181810 PMC11967198

