# Supplementary material for: Acute airway eosinophilic inflammation model in mice induced by ovalbumin, house dust mite, or shrimp tropomyosin: a comparative study
Source: Front Allergy. 2025 Jun 3;6:1594028. doi: 10.3389/falgy.2025.1594028 (PMC12170599; doi:10.3389/falgy.2025.1594028)
Supplement: Supplementary file 1 [file Table1.docx]

**Supplemantary Table 1 Primer sequences for qRT-PCR**

| **Gene** | **Forward primer (5'-3')** | **Reverse primer (5'-3')** |
| --- | --- | --- |
| mus-Il-4 | CATCGGCATTTTGAACGAG | CGAGCTCACTCTCTGTGGTG |
| mus-Il-5 | ACATTGACCGCCAAAAAGAG | CACCATGGAGCAGCTCAG |
| mus-Il-13 | ACCCAGAGGATATTGCATGG | TGGGCTACTTCGATTTTGGT |
| mus-Ccl-11 | GAATCACCAACAACAGATGCAC | ATCCTGGACCCACTTCTTCTT |
| mus-Ccl-17 | TACCATGAGGTCACTTCAGATGC | GCACTCTCGGCCTACATTGG |
| mus-Muc5ac | TCGAGAGGAGCGTTGACAC | GAGGGTTGCATTGAGGTCAT |
| mus-Ccl-2 | TAAAAACCTGGATCGGAACCAAA | GCATTAGCTTCAGATTTACGGGT |
| mus-Ccl-22 | AGGTCCCTATGGTGCCAATGT | CGGCAGGATTTTGAGGTCCA |
| mus-Ccl-24 | TCTTGCTGCACGTCCTTTATT | GCATCCAGTTTTTGTATGTGCC |
| mus-Cxcl-1 | ACTGCACCCAAACCGAAGTC | TGGGGACACCTTTTAGCATCTT |
| mus-Cxcl-10 | CCAAGTGCTGCCGTCATTTTC | TCCCTATGGCCCTCATTCTCA |
| mus-Cxcl-13 | ATATGTGTGAATCCTCGTGCCA | GGGAGTTGAAGACAGACTTTTGC |
| mus-Gapdh | CAAAATGGTGAAGGTCGGTGTG | TGATGTTAGTGGGGTCTCGCTC |

qRT-PCR, quantitative real-time PCR.
