# Supplementary figures and images for: Acute airway eosinophilic inflammation model in mice induced by ovalbumin, house dust mite, or shrimp tropomyosin: a comparative study
Source: Front Allergy. 2025 Jun 3;6:1594028. doi: 10.3389/falgy.2025.1594028 (PMC12170599; doi:10.3389/falgy.2025.1594028)

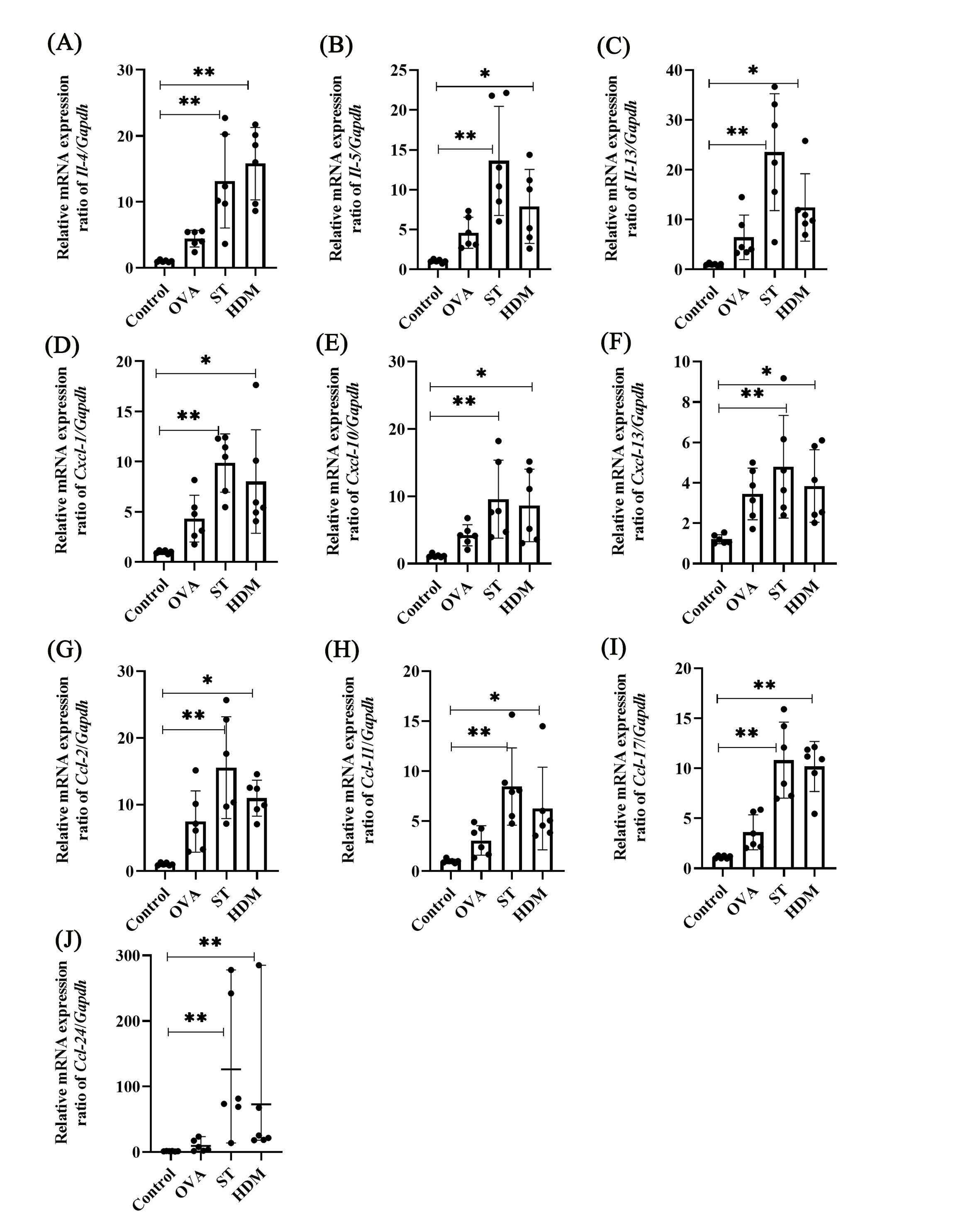

Supplement: Supplementary Figure S1 — Th2 cytokines and chemokine levels in OVA-, ST-, and HDM-treated mice. mRNA levels of Il-4 (A), Il-5 (B), Il-13 (C), Cxcl1 (D), Cxcl10 (E), Cxcl13 (F), Ccl2 (G), Ccl11 (H), Ccl17 (I), and Ccl24 (J) in the BAL-treated (lavaged) lung tissues (n = 6). Data represent mean ± SD. For Ccl-24 mRNA level, data are expressed as median ± range. *P < 0.05, **P < 0.01. [file Image1.jpeg]
